# Determination of Median Lethal Concentration (LC_50_) for Endosulfan, Heptachlor and Dieldrin Pesticides to African Catfish, *Clarias gariepinus* and Their Impact on Its Behavioral Patterns and Histopathological Responses

**DOI:** 10.3390/toxics9120340

**Published:** 2021-12-08

**Authors:** Md. Ariful Islam, S. M. Nurul Amin, Christopher L. Brown, Abdul Shukor Juraimi, Md. Kamal Uddin, Aziz Arshad

**Affiliations:** 1 Department of Aquaculture, Faculty of Agriculture, Universiti Putra Malaysia (UPM), Serdang 43400, Malaysia; arifulbau@gmail.com (M.A.I.); smnabd@gmail.com (S.M.N.A.); 2 Shrimp Research Station, Bangladesh Fisheries Research Institute (BFRI), Bagerhat 9300, Bangladesh; 3 Department of Aquaculture, FAO World Fisheries University, Busan 48547, Korea; brownchristopher38@gmail.com; 4 Department of Crop Science, Faculty of Agriculture, Universiti Putra Malaysia (UPM), Serdang 43400, Malaysia; ashukur@upm.edu.my; 5 Department of Land Management, Faculty of Agriculture, Universiti Putra Malaysia (UPM), Serdang 43400, Malaysia; mkuddin@upm.edu.my

**Keywords:** heptachlor, endosulfan, dieldrin, LC_50_, behavioral stresses, histopathological responses, African catfish (*Clarias gariepinus*)

## Abstract

Pesticides such as endosulfan, heptachlor and dieldrin persist in aquatic environments as a result of their resistance to biodegradation. However, there is no adequate information about the toxicity of endosulfan, heptachlor and dieldrin to the aquatic organism, African catfish (*Clarias gariepinus*)—a high valued widely distributed commercially interesting species. The current experiment was performed with the aim to determine the median lethal concentration (LC_50_) of endosulfan, heptachlor and dieldrin to African catfish (*Clarias gariepinus*); their behavioral abnormalities and histopathological alterations in several vital organs. A total of 324 juvenile fish were exposed for 96 h to six concentrations of endosulfan and dieldrin at 0, 0.001, 0.002, 0.004, 0.008 and 0.016 ppm, and to heptachlor at concentrations of 0, 0.02, 0.04, 0.08, 0.16 and 0.32 ppm for dose-response tests. The study demonstrated that the species is highly susceptible to those contaminants showing a number of behavioral abnormalities and histopathological changes in gill, liver and muscle. The 96-h LC_50_ value of endosulfan, dieldrin and heptachlor for the African catfish was found as 0.004 (0.001−0.01) mg/L, 0.006 mg/L and 0.056 (0.006−0.144) mg/L, respectively. Abnormal behaviors such as erratic jerky swimming, frequent surfacing movement with gulping of air, secretion of mucus on the body and gills were observed in response to the increasing exposure concentrations. Histopathological alterations of liver, gill and muscle tissues were demonstrated as vacuolization in hepatocytes, congestion of red blood cells (RBCs) in hepatic portal vein; deformed secondary lamellae and disintegrated myotomes with disintegrated epidermis, respectively. These findings are important to monitor and responsibly manage pesticide use in and around *C. gariepinus* aquacultural areas.

## 1. Introduction

Pesticides are widely applied in intensive agricultural and aquaculture settings to manage pest populations such as insects, weeds, mollusks and microbial pathogens. Pesticides can enter aquatic environments through direct applications or through several indirect mechanisms including runoff, drainage and wind drift [1], eventually affecting non-target aquatic organisms such as fish and crustaceans, thereby having considerable ecological and economic impacts [2]. Among commonly used pesticides, organochlorines are among the most serious concerns as a result of their persistence in air, sediment and ground water, and their propensity for bioaccumulation in food webs, often resulting in deposition in fish gill, liver, muscle, kidney, stomach and brain tissues [3,4,5].

These pesticides are associated with a range of carcinogenic, teratogenic and endocrine-disruptive effects in vertebrate organisms, including humans [6]. Organochlorine pesticides (OCPs) are categorized as ‘severely hazardous’ pesticides [7] and toxic to fish at moderate concentrations [8].

Endosulfan, Dieldrin and Heptachlor are highly problematic among organochlorine pesticides. Endosulfan was first registered in the USA during 1954 to manage agricultural mites, insects, and other pests [8]. Endosulfan is a highly persistent organic pollutant with a half-life in water from 3 to around 150 days, depending on the parameters such as pH, turbidity, dissolved oxygen and the presence or absence of aquatic pollutants [9]. Endosulfan is extensively applied in rice paddies in developing countries because of its relatively low cost and high degree of effectiveness, despite official prohibition of its use in developed countries such as the UK, the Netherlands, Sweden, Germany, Singapore and Columbia [10]. Endosulfan starts to exert toxic effects on aquatic organisms even at concentrations as low as 0.005 mg/L and hepatotoxic actions can occur quickly after absorption through skin, lung or stomach [10].

Dieldrin is used worldwide to control pests in agriculture and fish farming [11]. Dieldrin interacts at the neurotransmitter receptor level in the fish nervous systems, as reported in largemouth bass (*Micropterus salmoides*), causing neurotoxicity in the brain [12,13].

Heptachlor is an organochlorine pesticide extensively used to control termites and other insects in developed countries for the past 30 years [14]. Due to its toxicity to animals and humans, the U.S. and other developed countries have banned its use since 1978 [15]. However, heptachlor is still used for seed treatment and pre-planting agricultural practice by several tropical and subtropical countries [14]. Heptachlor is similar to other OCPs because of its chemical features and the compound persists in the soil or sediment for several years even at trace concentrations in the parts-per-billion (ppb) range [15]. The recorded half-life of heptachlor is around 250 days [16], although residues of heptachlor have been reported in soil 14 to 16 years after application, thereby resulting in groundwater pollution [14]. Oxidation during metabolism by a variety of plants and animals can generate heptachlor epoxide (a metabolite or degradation product of heptachlor) which by comparison is more toxic, and degrades relatively slower [15]. Heptachlor and heptachlor epoxide are both poorly soluble in water, leading to adsorption to sediments and bioaccumulation in aquatic food chains, particularly affecting benthic shellfishes and teleosts [9]. Fatty tissues, liver, muscle and neural structures of fish and shellfishes are vulnerable to heptachlor and heptachlor epoxide accumulation. These compounds have extended half-lives from months to years in lipid-based structures. Heptachlor and its metabolites are particularly toxic in liver [17]. Endosulfan concentration was reported in Snakehead (*Channa striatus*), Javanese carp (*Puntius gonionotus*), Gourami (*Trichogaster* sp.) and Climbing perch (*Anabas testudineus*) at 0.8–4.8, 1.1–4.8, 0.4–3.9 and 0.4–4.2 ng/g, respectively, from the Peninsular Malaysia rice fields [18]. Dieldrin concentration was observed in Catfish (*Arius* sp.), Blood cockle (*Anadara granosa*) and Mullet (*Valamugil* sp.) at 0.02–0.50, 0.01–0.70 as well as 0.02–0.8 ng/g, respectively, in the Straits of Malacca, Malaysia [18]. Heptachlor concentration was also found in Catfish (*Arius* sp.), Blood cockle (*Anadara granosa*) and Mullet (*Valamugil* sp.) at 0.3–8.2, 0.27–3.54 and 0.1–5.2 ng/g, respectively, from the same area [18]. These reports indicate the presence of endosulfan, dieldrin and heptachlor residues at the rice fields areas in Malaysia.

Fishes are sensitive indicators of environmental contaminants because of their consistent responsiveness to dilute pollutant exposure, many of which measurably disrupt physiological and biochemical mechanisms [19]. Histopathology contributes an efficient means of distinguishing the impact of pollutants in various organs such as a number of lesions in particular organs [20] such as gill [21] and liver [22]. These are considered convenient organs for histological observation to observe the effect of pollution. Other aquatic organisms contaminated with sublethal concentrations of pesticides may cause histological and physiological alterations in tissues of vital organs [23].

A few studies have been performed on how several endosulfan concentrations affect survival and behavioural abnormalities as well as histopathological responses in the Asian swamp eel, *Monopterus albus* and Climbing perch, *Anabas testudineus* from the Muda rice fields, Malaysia [24,25]. The study revealed that *M. albus* and *A. testudineus* are highly susceptible to endosulfan causing alarming effect on survival, behavioural abnormalities as well as histopathological alterations in several vital organs. However, the toxicity of endosulfan, dieldrin and heptachlor in African catfish, *Clarias gariepinus* (a commercially important species in aquaculture) remains poorly understood.

In Malaysia, *Clarias gariepinus* is the dominant fish species in rice producing regions, ranking as the second highest contributor to aquaculture production, following *Oreochromis niloticus*, the Nile Tilapia [26]. The culturing species may be threatened due to the considerable risk of exposure to agro-chemicals applied in crop-production areas with waste discharges that reach groundwater [27]. Hence, this study aims to improve our knowledge of how endosulfan, dieldrin and heptachlor affect African catfish, *Clarias gariepinus* through the determination of the median lethal concentration (LC_50_) values; investigation of the behavioral abnormalities as well as observation of the histopathological responses of gill, liver and muscle to these compounds.

## 2. Materials and Methods

This study was conducted at the Wet Lab, Department of Aquaculture, Faculty of Agriculture, Universiti Putra Malaysia (UPM), 43,400 UPM, Darul Ehsan, Selangor, Malaysia in full compliance with the ethics protocol of Institutional Animal Care and Use Committee (IACUC) of Universiti Putra Malaysia (UPM), as evaluated accordingly IACUC ethics approval reference no.: UPM/IACUC/AUP-R019/2021; Dated: 23 July 2021.

### 2.1. Fish and Acclimation Condition

A total of 665 juvenile (16–17 cm total length) African catfish were placed into a fiberglass tank (water holding capacity 1 ton) for 2 weeks prior to pesticide exposure. Dechlorinated tap water was used as the test medium. The fiberglass tank was outfitted with centralized aeration, using 4 aerated outlets inside the tank and a central drainage system for easy waste removal. Water temperature of the tank was consistent with the surrounding ambient temperature. Fish were given commercial floating pellets (SISO Goldy Color) twice daily to satiation level and approximately 40% of the water was exchanged daily for removal of fecal matters and leftover food particles to ensure optimal water quality [28]. The water quality parameters were maintained following the fish acute toxicity testing guidelines (OECD Test Guideline No. 203) [29]. During acclimation, the mortality rate was ≤2%. From the acclimatized juvenile stock, a total of 648 juveniles (324 juveniles for the range test and 324 juveniles for final test) were selected for determination of the median lethal concentration.

### 2.2. Preparation of Aquariums and Stocking of Tests Fish

In this case, 18 aquariums (76 cm × 38 cm × 38 cm) with 40 L capacity were prepared for experimental exposure to each pesticide, with three units for each concentration. For avoidance of the fungal infection, the glass aquaria were cleaned with 1% potassium permanganate (KMnO_4_) and therefore sun dried before the exposure tests. Before stocking, fish were screened for any pathogenic infections. The active and healthy fish were placed into the glass aquariums containing dechlorinated tap water. Six (06) individuals were released in each aquarium to avoid the development of maladaptive behavior to improve animal welfare [30]. The aquariums were equipped with aeration system to ensure optimum oxygen level [31] where 12 h light and 12 h dark (LD 12:12) photoperiod was maintained [29].

### 2.3. Exposure of Contaminants and Determination of LC_50_ (Range Test and Final Test)

The exposure test was a static (i.e., the pesticides concentrations were kept constant) based test where endosulfan and dieldrin based pesticide solutions were made with a concentration of 0 (no pesticides, only n-hexane solvent mixed here as control), 0.0001, 0.001, 0.01, 0.1 and 1 mg/L for range test. In the range test of endosulfan exposure, 100% mortality of test fish occurred within 24-h when the concentration was 1 mg/L. The mortality was 33% of the test fish at the concentration of 0.001 mg/L and at the concentration level of 0.01 mg/L, the mortality rate of the test fish was 67%. So, the final experiment for median lethal concentration of endosulfan exposure was carried out at concentration levels ranging from 0.001 to 0.01 mg/L. The range test results showed that the LC_50_ of endosulfan was within 0.001 to 0.019 mg/L which is presented in Table 1. In the range test for dieldrin exposure, 100% mortality of the test fish happened within 24-h at the concentration of 1 mg/L. Percentage mortality of test fish in range test for the different concentrations of dieldrin is given in Table 2. The dieldrin concentration level of 0.001 mg/L caused 33% mortality of the test fish and the concentration 0.1 mg/L obtained 83% average mortality of the test fish (*Clarias gariepinus*). So, the concentration levels within 0.001 to 0.1 mg/L were taken into consideration for the final test to determine the median lethal concentration of dieldrin exposure to the test fish (*C. gariepinus*). The range test reported that the median lethal concentration (LC_50_) of dieldrin was within 0.001 to 0.032 mg/L.

Then for the final test of endosulfan and dieldrin the solutions were made with a concentration of 0 (no pesticides, only n-hexane solvent mixed here as control), 0.001, 0.002, 0.004, 0.008 and 0.016 mg/L [32].

Heptachlor based pesticide solution was prepared with a concentration of 0 (no pesticides, only n-hexane solvent mixed here as control), 0.002, 0.02, 0.2, 1 and 2 mg/L for the range test. In the range test of heptachlor, the concentration of 2 mg/L resulted 100% mortality of test fish within 24 h. Exposure to the concentration of 0.02 mg/L resulted 33% mortality of the test fish, and at the concentration level of 1 mg/L, 83% mortality rate of the test fish was observed within 96 h exposure (Table 3). So, the final test for median lethal concentration of heptachlor was carried out considering the concentration levels ranged from 0.02 to 1 mg/L. The range test result demonstrated that the LC_50_ value of heptachlor was within 0.005 to 0.207 mg/L.

After that pesticide solutions were made with a concentration of 0 (no pesticides, only n-hexane solvent mixed here as control), 0.02, 0.04, 0.08, 0.16 and 0.32 mg/L for the final test [32]. For each pesticide treatment, the individual concentration was subjected to three replications. The analytical grade endosulfan, dieldrin and heptachlor were exposed in this experiment which obtained from SIGMA-Aldrich, Germany through JM Instrument and Chemical Supply, Kajang, Selangor, Malaysia. The purity of endosulfan, dieldrin and heptachlor was more than 98–99%. For preparing the stock solution of the pesticides, 1 mL n-hexane was used to dissolve the pesticides homogenously. During the exposure of pesticide, no feeding was carried out [32]. The exposure period was 96-h for the test fish, *Clarias gariepinus.* Fish were examined after 2 ± 0.5 h, 5 ± 1 h and 24 ± 2 h from the exposure of pesticides (day 0–1). During the days 2–4 of the examination, all aquaria with living fish were investigated twice per day at early morning and late afternoon. The mortality rate was observed as the number of dead test fish once every 12 h, and then calculated on a cumulative basis for 96-h [29]. The mortality percentage was calculated from the number of dead fish divided by the total number of test fish for each treatment level [33].

Critical range tests were carried out to determine the concentrations which causing 50% mortality as used in the final test [30].

### 2.4. Water Quality during Experiments

Water quality was ensured throughout the experiment. Dissolved oxygen (DO), temperature, pH, electrical conductivity (EC) and total dissolved solid (TDS) of the test media (water) were monitored using Multimeter (YSI) to ensure optimal water quality [32]. The water quality parameters are presented in Table 4 which were monitored after 24-h of exposure of the endosulfan, dieldrin and heptachlor.

Although temperature and pH play a vital role on the toxicity performance of pesticides [20,34,35,36], in our study the water quality parameters remained within acceptable limits and only toxic effects of the pesticides were apparent here.

### 2.5. Investigation of the Behavioural Abnormalities of Fish

The behavioral alterations of the test fish were examined during the exposure of endosulfan, dieldrin and heptachlor for 24–96 h. The behavioral alterations are associated with the physiological responses which are indicative of stress [37]. Pesticide exposure not only results in mortality, but may also result in behavioral abnormalities at sub-lethal pesticide concentrations [30,38]. The behavioral abnormalities of fish such as hyperactivity, jerky movement, abnormal swimming behavior, loss of equilibrium, abnormal ventilatory function, mucus secretion and abnormal skin pigmentation were monitored at a six hours interval through visual inspection and video recordings during the 96-h exposure of heptachlor, dieldrin and endosulfan (OECD Test Guideline No. 203) [29]. The behavioral abnormalities were categorized as ‘mild’, ‘moderate’ and ‘severe’ where ‘mild’ indicated 30 to 35% of individuals showed the abnormalities; ‘moderate’ indicated 45 to 50% of individuals showed the abnormalities) and ‘severe’ indicated 60 to ≥70% of individuals showed the behavioural abnormalities, respectively [11].

### 2.6. Study of Histopathological Responses

At the end of pesticide exposure period, tissues were dissected from moribund fish resulted from the exposure of LC_50_ dose only and processed for histopathological examination. Control tissues were dissected from the live fish of control aquarium. Samples from the liver, muscle (muscle was dissected from the skin flap under the dorsal fin) and gills were fixed in 10% neutral buffer formalin and were stained with hematoxylin and eosin [39]. The resolutions of the picture were adjusted at 40× magnification. The slides were examined and the histopathological alterations of hepatocytes, sinusoids, hepatic portal vein and red blood cells (RBCs) for the liver; epithelial cells, primary lamellae and secondary lamellae for the gill; epidermis, myotomes and septum for the muscle tissues were captured through light microscope, Motic-BA410, USA (obtained from San Antonio, Schertz, TX, USA) equipped with camera (Moticam pro) and the software MIP (Microscopic Image Processing).

### 2.7. Statistical Analysis

The 96-h LC_50_ values were estimated with Probit Analysis, using SPSS (version 20.0; SPSS, Chicago, IL, USA) through the dose-response relationship of fish mortality due to the exposure to endosulfan, dieldrin and heptachlor.

## 3. Results

### 3.1. Median Lethal Concentration (LC_50_) for 96 h Exposure to Endosulfan through Dose-Response Test

The dose-response test found no mortality at control (0 mg/L) during 96-h exposure period. Percentage mortality of test fish in different concentrations of endosulfan is presented in Table 5. The resulting 96-h LC_50_ value for endosulfan was 0.004 mg/L which resulted in 50% mortality of the test fish.

### 3.2. Median Lethal Concentration (LC_50_) for 96 h Exposure to Dieldrin through Dose-Response Test

In the dose-response test, no mortality was found at control (0 ppm) during 96-h exposure period. The percentage mortality of test fish in the dose-response test of dieldrin is presented in Table 6. After 96-h exposure to dieldrin, the resulting LC_50_ value was 0.006 mg/L.

### 3.3. Median Lethal Concentration (LC_50_) for 96-h Exposure to Heptachlor through Dose-Response Test

The *dose-response* test revealed that there was no mortality at control (0 mg/L) during the 96-h exposure period. Percentage mortality of test fish against different concentrations of heptachlor is displayed in Table 7. The resulting 96-h LC_50_ value for heptachlor was 0.057 mg/L.

### 3.4. Behavioural Abnormalities of Test Fish during the Exposure to Pesticides

No behavioral abnormalities were observed in the control fish. Fish treated with endosulfan at the concentration level from 0.001 to 0.016 mg/L (presented in Table 8), exposed to dieldrin at the concentration level from 0.001 to 0.016 mg/L (presented in Table 9) as well as fish exposed to heptachlor at a concentration level from 0.02 to 0.32 mg/L (demonstrated in Table 10) exhibited mild (around 30 to 35% of individuals showing abnormalities) to moderate (around 45 to 50% of individuals showing abnormalities) behavioral responses for the initial 48 h but afterwards fish started to show severe (around 60 to ≥70% of individuals showing abnormalities) behavioral reactions in terms of hyperactivity, jerky movement, abnormal swimming, disability of equilibrium, abnormalities in ventilatory function, mucus secretion as well as abnormalities in skin pigmentation. In most cases, fish treated to higher concentrations of the pesticides displayed severe abnormal behavior such as very fast swimming, jumping and displaying instability with severe jerky movements, speedy opercular movement, hyperexcitation, and surfacing with gulping of air.

### 3.5. Histopathological Responses of Liver, Gill and Muscle Tissues of Clarias gariepinus Due to the 96-h Exposure to Endosulfan, Dieldrin and Heptachlor

#### 3.5.1. Histopathological Transformations in Liver

**Figure 1:**
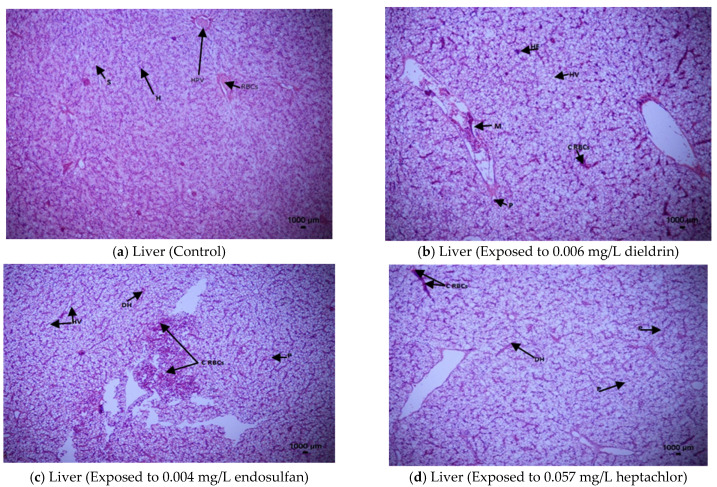
Histopathological alterations in the liver tissue of *Clarias gariepinus* at 96-h. exposure of endosulfan, dieldrin and heptachlor compared to control (unexposed) using H and E 40× magnification. (**a**) Liver (Control): H: Hepatocytes (polygonal in shape with a prominent nucleus), S: Sinusoids (fenestrated), HPV: Hepatic Portal Vein, RBCs: (red blood cells). (**b**). Liver (Exposed to 0.006 mg/L dieldrin): HV: Vacuolization in hepatocytes, HF: Hepatocytes fusion, M: Melano-macrophages center, P: Pyknotic nuclei, CRBCs: Congestion of RBCs in HPV of hepatocyte. (**c**). Liver (Exposed to 0.004 mg/L endosulfan): HV: Vacuolization in hepatocytes, DH: Disintegration of hepatocytes cell membrane and oozing of cytoplasmic content, P: Pyknotic nuclei, CRBCs: Congestion of RBCs in HPV of hepatocyte. (**d**). Liver (Exposed to 0.057 mg/L heptachlor): DH: Disintegration of hepatocytes cell membrane and oozing of cytoplasmic content, P: Pyknotic nuclei, CRBCs: Congestion of RBCs in HPV of hepatocyte.

The histopathological transformations in the liver of African catfish due to the 96-h exposure to endosulfan, dieldrin and heptachlor are summarized in Figure 1a–d. Fish in the control group (unexposed) showed typical structures of liver including hepatocytes (polygonal in shape with a prominent nucleus), sinusoids (fenestrated), regular hepatic portal vein and red blood cells [Figure 1a]. Vacuolization in hepatocytes, hepatocytes fusion, melano-macrophages center, pyknotic nuclei along with congestion of RBCs (red blood cells) in HPV (hepatic portal vein) of hepatocytes are reported for the exposure to dieldrin (0.006 mg/L) after 96 h [Figure 1b]. Vacuolization in hepatocytes, disintegration of hepatocytes cell membrane with oozing of cytoplasmic content, pyknotic nuclei as well as congestion of RBCs (red blood cells) in HPV (hepatic portal vein) of hepatocytes are recorded during the 96-h exposure to endosulfan (0.004 mg/L) [Figure 1c]. Congestion of RBCs (red blood cells) in HPV (hepatic portal vein) of hepatocytes, pyknotic nuclei and disintegration of hepatocytes cell membrane along with oozing of cytoplasmic content were observed after 96-h exposure to heptachlor (0.057 mg/L) in *Clarias gariepinus* [Figure 1d].

#### 3.5.2. Histopathological Responses in Gill

**Figure 2:**
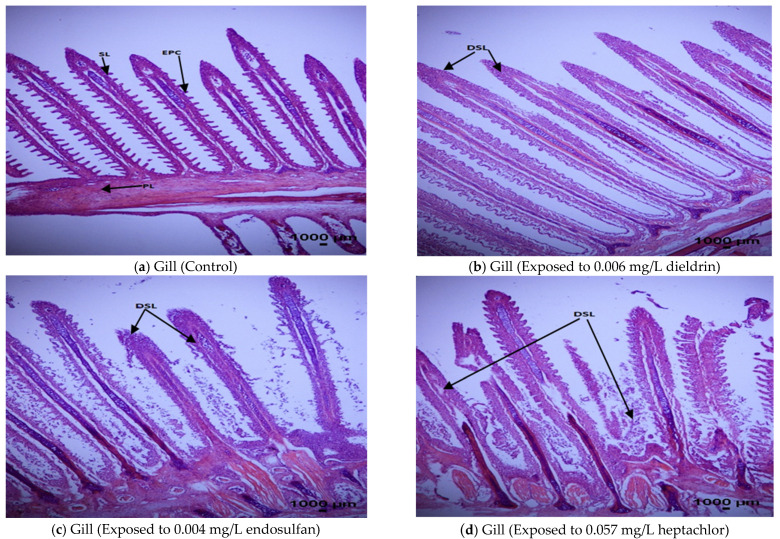
Histopathological alterations in the gill tissue of *Clarias gariepinus* at 96-h exposure of endosulfan, dieldrin and heptachlor compared to control (unexposed) using H and E 40× magnification. (**a**) Gill (Control): EPC: Epithelial cell, SL: Secondary lamellae, PL: Primary lamellae. (**b**) Gill (Exposed to 0.006 mg/L dieldrin): DSL: Deformed secondary lamellae. (**c**) Gill (Exposed to 0.004 mg/L endosulfan): DSL: Deformed secondary lamellae and Figure 2d. Gill (Exposed to 0.057 mg/L heptachlor): DSL: Deformed secondary lamellae.

The histopathological alterations in the gills of African catfish are illustrated in Figure 2a–d. after 96-h exposure to endosulfan, dieldrin and heptachlor. Fish in the unexposed group (control) observed normal structure in the epithelial cell, primary lamellae and secondary lamellae [Figure 2a]. Moderate deformation and loss of secondary lamellae happened during the exposure to dieldrin (0.006 mg/L) after 96-h [Figure 2b]. Severe deformation and loss of secondary lamellae occurred due to the exposure of endosulfan (0.004 mg/L) and heptachlor (0.057 mg/L) after 96-h [Figure 2c,d].

#### 3.5.3. Histopathological Responses in Muscle

**Figure 3:**
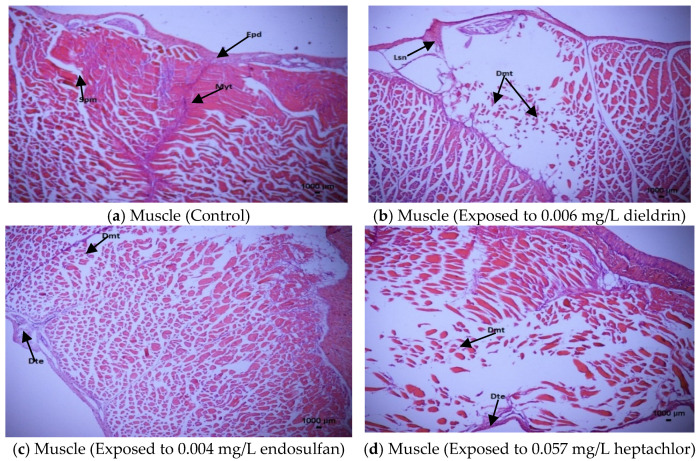
Histopathological alterations in the muscle tissue of *Clarias gariepinus* at 96-h exposure of endosulfan, dieldrin and heptachlor compared to control (unexposed) using H and E 40× magnification. (**a**) Muscle (Control): Epd: Epidermis, Myt: Myotomes, Spm: Septum. (**b**) Muscle (Exposed to 0.006 mg/L dieldrin): Dmt: Disintegrated myotomes, Lsn: Lesions. (**c**) Muscle (Exposed to 0.004 mg/L endosulfan): Dmt: Disintegrated myotomes, Dte: Disintegrated epidermis. (**d**) Muscle (Exposed to 0.057 mg/L heptachlor): Dmt: Disintegrated myotomes, Dte: Disintegrated epidermis.

During the 96-h exposure to endosulfan, dieldrin and heptachlor, the changes in the muscle tissues of African catfish are presented in Figure 3a–d. Fishes in the control group (unexposed) revealed typical structures of muscle tissue including normal epidermis, myotomes and septum [Figure 3a] whereas seriously disintegrated myotomes, disintegrated epidermis and noticeable lesions are found in the muscle tissues after 96-h exposure of dieldrin (0.006 mg/L) [Figure 3b] and heptachlor (0.057 mg/L) [Figure 3d]. Moderate disintegration of myotomes and epidermis are observed in the muscle tissues after 96-h exposure of endosulfan (0.004 mg/L) [Figure 3c].

## 4. Discussion

### 4.1. Median Lethal Concentration (LC_50_) for 96-h Exposure to Endosulfan, Dieldrin and Heptachlor

The 96-h LC_50_ value (0.004 mg/L) of endosulfan for African catfish determined in the present study is less than the values of 0.035 mg/L for *Anabas testudineus* [25], 0.024 mg/L for *Channa punctatus* [40], 0.01 to 0.013 mg/L for Nile tilapia, *Oreochromis niloticus* [31,41], 0.0078 mg/L for Silver perch, *Bidyanus bidyanus* [42] and 0.041 mg/L for European eel, *Anguilla anguilla* [43].

In contrast, the 96-h LC_50_ value (0.004 mg/L) of endosulfan for African catfish determined in the present study is higher than the LC_50_ for Rainbow trout, *Oncorhynchus mykiss* (0.0016 to 0.0018 mg/L) [20,42], Asian swamp eel, *Monopterus albus* (0.0004 mg/L) [24], Perciformes, *Cichlasoma dimerus* (0.0026 mg/L) [44], Tilapia, *Oreochromis mossambicus* (0.0036 mg/L) [45], Tilapia fingerling, *Oreochromis mossambicus* (0.0014 mg/L) [46] and European carp, *Cyprinus carpio* (0.002 mg/L) [47].

It was observed that the 96-h LC_50_ values for both dieldrin and heptachlor from the present study are consistent with the values from the studies presented in Table 11.

It is worthy of mention that LC_50_ is dependent on diverse factors such as the method of acute toxicity test, purity percentage of the exposure contaminants and the size and health status of fish used in the toxicity tests. For instance, [20,42] reported LC_50_ values of 0.0016 and 0.0018 mg/L for Rainbow trout using static and semi-static toxicity test, respectively. Contrarily, [45,46] obtained LC_50_ values of 0.0036 and 0.0014 mg/L for Tilapia of unequal sizes; i.e., 46.78 g fish and fingerlings, respectively. Environmental factors such as temperature, pH, alkalinity and turbidity were also reported to correlate with endosulfan toxicity [51]. For example, a pH of less than 5 would increase hydrolyzation of endosulfan to endosulfan sulphate, which is relatively more toxic [52]. LC_50_ of Rainbow trout depend on temperature and water quality parameters such as pH, alkalinity and hardness in addition to the fish sizes [20]. Moreover, organisms are usually exposed to several stressors at the same time that may interact with each other and lead to the different synergistic effects [53,54].

### 4.2. Behavioural Abnormalities of Test Fish during the Exposure to Pesticides

In our study, we observed several behavioral abnormalities of African catfish in response to acute exposure to endosulfan, dieldrin and heptachlor. The reported behavioral alterations such as hyperactivity, increased erratic swimming, excessive mucus secretion, loss of equilibrium, increase jerky movement, gasping and decreased fin movement are similar to patterns observed in zebrafish, *Danio rerio* [55] following exposure to endosulfan; in *Clarias gariepinus* [33] for the treatment of chloropyrifos; in *Channa punctatus* [56,57] with the exposure of cypermethrin; in *Cyprinus carpio* [58] by profenofos; in *Clarias gariepinus* [59] for malathion treatment as well as in Rainbow trout, *Oncorhynchus mykiss* [60] due to the exposure of carbosulfan pesticide. Behavioural abnormalities reduce the ability of fish to adequately respond to several environmental stimuli, which may for instance lead to lower foraging success and a higher susceptibility to predation [34,36].

### 4.3. Histopathological Responses of Liver, Gill and Muscle Tissues of Clarias gariepinus Due to the 96-h Exposure to Endosulfan, Dieldrin and Heptachlor

In our experiment, histology of liver of *Clarias gariepinus* exposed to endosulfan (0.004 mg/L), dieldrin (0.006 mg/L) and heptachlor (0.057 mg/L) revealed pyknotic nuclei with congestion of RBCs (red blood cells) in HPV (hepatic portal vein) of hepatocytes, hepatocytes fusion, vacuolization in hepatocytes, melano-macrophages canter and disintegration of hepatocytes cell membrane with oozing of cytoplasmic content. Our findings are comparable with the histological alterations of liver in *Puntius conchonius* [61], *Oncorhynchus mykiss* [60], *Cichlasoma dimerus* [44], *Oreochromis mossambicus* [45], *Heteropneustes fossilis* [62] exposed to endosulfan and dieldrin such as organochlorine pesticides. The present study showed that there is a strong link between liver damage and toxicants. Histological anomalies observed in fish liver tissue after acute exposure of organochlorine pesticides can cause functional disabilities resulting in malfunctioning of various organ systems [60].

In our study, histopathology of gill of *C. gariepinus* exposed to endosulfan (0.004 mg/L), dieldrin (0.006 mg/L) and heptachlor (0.057 mg/L) presented thickening of primary lamellae epithelium, shorting of secondary lamellae, epithelial hyperplasia and lamellar fusion (fusion of secondary lamellae) with thickening of primary lamellar epithelium along with collapsed secondary lamellae. To validate our findings, similar histological alterations are also found in the gill of *Chanos chanos* [63], *Oncorhynchus mykiss* [60], *Cichlasoma dimerus* [44], *Oreochromis mossambicus* [64], *Salmo salar* [65], *Channa punctatus* [66] as well as *Hyphessobrycon bifasciatus* and *Danio rerio* [67] after acute exposure to endosulfan, as with other organochlorine pesticides. Exposure of fish to pollutants such as pesticides collapses gills which may disrupt gas exchange efficiency resulting in respiratory disorders, osmoregulatory dysfunction and ion-regulation imbalance to force the excretion of nitrogenous wastage products [68].

The histopathological transformations in the muscle of African catfish, *Clarias gariepinus* are in agreement with the histological alterations in zebrafish, *Danio rerio* [69], *Hoplias alabaricus* [70], *Mugil capito* [71] as well as redbelly Tilapia, *Tilapia zillii* and Common sole, *Solea vulgaris* [72].

The liver plays vital physiological functions such as detoxification of xenobiotics, synthesis of components of the blood, glycogen storage along with release of glucose in the blood [73]. Fish gills provide essential functions such as gas exchange, ion transportation, nitrogenous wastage excretion as well as uptake and excretion of particular xenobiotics [74]. In spite of having detoxifying capacity, the balancing system of liver may be collapsed showing its structural disruption due to the enhanced concentration of hazardous compounds [75].

Based on the LC_50_ values obtained from this study, it is clear that *Clarias gariepinus* is highly susceptible to the effect of endosulfan, dieldrin and heptachlor. Therefore, these pesticides could cause severe behavioural abnormalities and serious structural changes of the vital organs, i.e., liver and gill of the fish which threaten their population in the rice field ecosystem.

## 5. Conclusions

This work investigates the acute toxicity of endosulfan, dieldrin and heptachlor to African catfish *Clarias gariepinus* through dose-response relationship, including also behavioral abnormalities and histopathological alterations of vital organs. The toxicity was observed to increase with the concentration of endosulfan, dieldrin and heptachlor. The behavioral abnormalities were observed with the increasing concentration of endosulfan, dieldrin and heptachlor. Particular structural changes were also observed in the vital organs, i.e., gill, liver as well as muscle tissue with the exposure to median lethal concentration of endosulfan, dieldrin and heptachlor. The histopathological and behavioral changes at sublethal levels could indirectly lead to lower survival and reproduction at the individual level and in this way disrupt the population dynamics of *C. gariepinus.* The fish could also bioconcentrate the pesticides and thus become a hazard to human consumers.

## Figures and Tables

**Table 1:** Range test for (%) mortality response of test fish to the exposure of endosulfan.

Exposure Conc. (mg/L)	Initial No. of Test Fish	Count of Dead Fish with the Time of Exposure				Cumulative Count of Dead Fish within 96-h. Exposure Time	% Mortality Response (avg.)
		24 h	48 h	72 h	96 h		
0	18	0	0	0	0	0	0
0.0001	18	0	0	0	3	3	17
0.001	18	0	0	0	6	6	33
0.01	18	0	3	3	6	12	67
0.1	18	0	12	3	0	15	83
1	18	18	0	0	0	18	100

**Table 2:** Range test for (%) mortality response of test fish to the exposure of dieldrin.

Exposure Conc. (mg/L)	Initial No. of Test Fish	Count of Dead Fish with the Time of Exposure				Cumulative Count of Dead Fish within 96-h Exposure Time	% Mortality Response (avg.)
		24 h	48 h	72 h	96 h		
0	18	0	0	0	0	0	0
0.0001	18	0	0	0	3	3	17
0.001	18	0	0	3	3	6	33
0.01	18	0	0	3	6	9	50
0.1	18	6	6	3	0	15	83
1	18	18	0	0	0	18	100

**Table 3:** Range test for (%) mortality response of test fish to the exposure of heptachlor.

Exposure Conc. (mg/L)	Initial No. of Test Fish	Count of Dead Fish with the Time of Exposure				Cumulative Count of Dead Fish within 96-h Exposure Time	% Mortality Response (avg.)
		24 h	48 h	72 h	96 h		
0	18	0	0	0	0	0	0
0.002	18	0	0	0	3	3	17
0.02	18	0	0	3	3	6	33
0.2	18	0	0	6	6	12	67
1	18	6	6	3	0	15	83
2	18	18	0	0	0	18	100

**Table 4:** Physico-chemical characteristics of water during the experiment.

Compounds	Conc. (mg/L)	Tem. (°C)	DO (mg O_2_/L)	pH	TDS (mg/L)	EC (mS/cm)
Endosulfan	0	25.60 ± 0.10	6.15 ± 0.02	6.63 ± 0.03	0.301	0.193
	0.001	25.60 ± 0.20	6.14 ± 0.01	6.68 ± 0.33	0.301	0.193
	0.002	25.74 ± 0.16	6.10 ± 0.04	6.73 ± 0.38	0.301	0.193
	0.004	25.79 ± 0.22	6.09 ± 0.02	6.75 ± 0.30	0.301	0.193
	0.008	25.84 ± 0.13	6.05 ± 0.01	6.78 ± 0.30	0.301	0.192
	0.016	25.85 ± 0.26	5.93 ± 0.05	6.84 ± 0.14	0.301	0.192
Dieldrin	0	25.50 ± 0.15	6.21 ± 0.02	6.65 ± 0.03	0.299	0.186
	0.001	25.60 ± 0.20	6.17 ± 0.03	6.68 ± 0.13	0.299	0.188
	0.002	25.64 ± 0.16	6.16 ± 0.04	6.76 ± 0.21	0.299	0.189
	0.004	25.76 ± 0.20	6.13 ± 0.02	6.78 ± 0.30	0.300	0.192
	0.008	25.78 ± 0.12	6.09 ± 0.01	6.80 ± 0.03	0.301	0.193
	0.016	25.86 ± 0.23	5.98 ± 0.02	6.83 ± 0.12	0.301	0.193
Heptachlor	0	25.65 ± 0.12	6.18 ± 0.02	6.65 ± 0.05	0.296	0.183
	0.02	25.68 ± 0.02	6.16 ± 0.01	6.67 ± 0.32	0.296	0.187
	0.04	25.72 ± 0.06	6.12 ± 0.03	6.71 ± 0.18	0.297	0.190
	0.08	25.78 ± 0.12	6.09 ± 0.05	6.75 ± 0.30	0.300	0.191
	0.16	25.82 ± 0.16	6.08 ± 0.04	6.77 ± 0.03	0.301	0.193
	0.32	25.87 ± 0.27	5.95 ± 0.05	6.81 ± 0.11	0.301	0.193

**Table 5:** Median lethal concentration (LC_50_) of endosulfan after 96-h exposure to the test fish.

Exposure Conc. (mg/L)	Total Test Fish	No. of Dead Fish			Total No. of Dead Fish	% Mortality	LC_50_ (mg/L)
		Replicate					
		1	2	3			
0	18	0	0	0	0	0	0.004 (0.001−0.01)
0.001	18	2	1	3	6	33	
0.002	18	2	3	2	7	38	
0.004	18	4	2	3	9	50	
0.008	18	3	4	4	11	61	
0.016	18	4	5	4	13	72	

During the Probit analysis, Chi-Square value, *χ*^2^ was 0.095 and no heterogeneity factor was observed at 95% confidence limits. Coefficient of determination value, *R*^2^ was 0.98 as well as the lower confidence limit (LCL) was 0.001 and upper confidence limit (UCL) was 0.01, at 95% confidence limit.

**Table 6:** Median lethal concentration (LC_50_) of dieldrin after 96-h exposure to the test fish.

Exposure Conc. (mg/L)	Total Test Fish	No. of Dead Fish			Total No. of Dead Fish	% Mortality	LC_50_ mg/L)
		Replicate					
		1	2	3			
0	18	0	0	0	0	0	0.006
0.001	18	2	1	3	6	33	
0.002	18	3	2	2	7	38	
0.004	18	2	3	3	8	45	
0.008	18	3	4	2	9	50	
0.016	18	4	4	3	11	62	

During the Probit analysis, Chi-Square value, *χ*^2^ was 0.084 and no heterogeneity factor was observed at 95% confidence limits, and the Coefficient of determination value, *R*^2^ was 0.98.

**Table 7:** Median lethal concentration (LC_50_) of heptachlor after 96-h exposure to the test fish.

Exposure Conc. (mg/L)	Total Test Fish	No. of Dead Fish			Total No. of Dead Fish	% Mortality	LC_50_ (mg/L)
		Replicate					
		1	2	3			
0	18	0	0	0	0	0	0.056 (0.006−0.144)
0.02	18	2	1	3	6	33	
0.04	18	2	3	4	9	50	
0.08	18	3	4	3	10	55	
0.16	18	4	4	3	11	61	
0.32	18	4	5	4	13	72	

During the Probit analysis, Chi-Square value, *χ*^2^ was 0.283 and no heterogeneity factor was observed at 95% confidence limits. The Coefficient of determination value, *R*^2^ was 0.96 as well as the lower confidence limit (LCL) was 0.006 and upper confidence limit (UCL) was 0.144, at 95% confidence limit.

**Table 8:** Behavioural abnormalities of *Clarias gariepinus* due to the exposure to endosulfan within the 96-h observation period.

Conc. (mg/L).	Hyper Activity	Jerky Movement	Abnormal Swimming Behaviour	Loss of Equilibrium	Abnormal Ventilatory Function	Mucus Secretion	Abnormal Skin Pigmentation
24 h							
Control	-	-	-	-	-	-	-
0.001	-	-	+	-	-	-	-
0.002	+	-	+	-	-	-	-
0.004	+	-	+	-	+	-	-
0.008	+	+	+	+	+	-	-
0.016	+	+	++	+	+	-	-
48 h							
Control	-	-	-	-	-	-	-
0.001	+	+	+	-	-	-	-
0.002	+	-	+	-	+	-	-
0.004	+	+	+	+	+	-	-
0.008	++	+	+	+	+	+	-
0.016	++	++	++	++	++	++	+
72 h							
Control	-	-	-	-	-	-	-
0.001	+	+	+	+	+	+	-
0.002	++	+	+	+	+	+	-
0.004	++	++	++	++	++	+	+
0.008	++	++	++	++	++	++	++
0.016	+++	+++	+++	+++	+++	+++	++
96 h							
Control	-	-	-	-	-	-	-
0.001	+	+	+	+	+	+	+
0.002	++	++	++	++	++	++	+
0.004	++	++	++	++	++	++	++
0.008	+++	+++	+++	+++	+++	+++	++
0.016	+++	+++	+++	+++	+++	+++	+++

‘-’ indicates no behavioural abnormality; ‘+’ indicates number of individuals showing the behavioural abnormalities; ‘+’ = Mild (around 30 to 35% of individuals showing the behavioural abnormalities); ‘++’ = Moderate (around 45 to 50% of individuals showing the behavioural abnormalities) and ‘+++’ = Severe (around 60 to ≥70% of individuals showing the behavioural abnormalities).

**Table 9:** Behavioural abnormalities of *Clarias gariepinus* due to the exposure to dieldrin within the 96-h observation period.

Conc. (mg/L).	Hyper Activity	Jerky Movement	Abnormal Swimming Behaviour	Loss of Equilibrium	Abnormal Ventilatory Function	Mucus Secretion	Abnormal Skin Pigmentation
24 h							
Control	-	-	-	-	-	-	-
0.001	-	-	+	-	-	-	-
0.002	+	-	+	-	-	-	-
0.004	+	-	+	-	+	-	-
0.008	+	+	+	+	+	-	-
0.016	+	+	++	+	+	-	-
48 h							
Control	-	-	-	-	-	-	-
0.001	+	+	+	-	-	-	-
0.002	+	-	+	-	+	-	-
0.004	+	+	+	+	+	-	-
0.008	++	+	+	+	+	+	-
0.016	++	++	++	++	++	++	+
72 h							
Control	-	-	-	-	-	-	-
0.001	+	+	+	+	+	+	-
0.002	++	+	+	+	+	+	-
0.004	++	++	++	++	++	+	+
0.008	++	++	++	++	++	+	+
0.016	++	++	++	++	++	++	++
96 h							
Control	-	-	-	-	-	-	-
0.001	+	+	+	+	+	+	+
0.002	++	++	++	++	++	+	+
0.004	++	++	++	++	++	++	++
0.008	+++	++	++	+++	++	++	++
0.016	+++	+++	+++	+++	+++	+++	++

‘-’ indicates no behavioural abnormality; ‘+’ indicates number of individuals showing abnormalities; ‘+’ = Mild (around 30 to 35% of individuals showing the behavioural abnormalities); ‘++’ = Moderate (around 45 to 50% of individuals showing the behavioural abnormalities) and ‘+++’ = Severe (around 60 to ≥70% of individuals showing the behavioural abnormalities).

**Table 10:** Behavioural abnormalities of *Clarias gariepinus* due to the exposure to heptachlor within the 96-h observation period.

Conc. (mg/L).	Hyper Activity	Jerky Movement	Abnormal Swimming Behaviour	Loss of Equilibrium	Abnormal Ventilatory Function	Mucus Secretion	Abnormal Skin Pigmentation
24 h							
Control	-	-	-	-	-	-	-
0.02	-	-	+	-	-	-	-
0.04	+	-	+	-	-	-	-
0.08	+	-	+	-	+	-	-
0.16	+	+	+	+	+	-	-
0.32	+	+	++	+	+	-	-
48 h							
Control	-	-	-	-	-	-	-
0.02	+	+	+	-	-	-	-
0.04	+	-	+	-	+	-	-
0.08	+	+	+	+	+	-	-
0.16	++	+	+	+	+	+	-
0.32	++	++	++	++	++	++	+
72 h							
Control	-	-	-	-	-	-	-
0.02	+	+	+	+	+	+	-
0.04	++	+	+	+	+	+	-
0.08	++	++	++	++	++	+	+
0.16	++	++	++	++	++	++	++
0.32	+++	+++	+++	+++	+++	+++	++
96 h							
Control	-	-	-	-	-	-	-
0.02	+	+	+	+	+	+	+
0.04	++	+	+	+	+	+	+
0.08	++	++	++	++	++	++	++
0.16	++	++	++	+++	++	++	++
0.32	+++	+++	+++	+++	+++	+++	++

‘-’ indicates no behavioural abnormality; ‘+’ indicates number of individuals showing the behavioural abnormalities; ‘+’ = Mild (around 30 to 35% of individuals showing abnormalities); ‘++’ = Moderate (around 45 to 50% of individuals showing the behavioural abnormalities) and ‘+++’ = Severe (around 60 to ≥70% of individuals showing the behavioural abnormalities).

**Table 11:** Comparison of LC_50_ for the toxicity of dieldrin and heptachlor pesticides exposed to different fish species.

Contaminant Pesticide	Fish Species	Life Stage	Test Type	Test Duration	LC_50_ (mg/L)	SOURCE
Dieldrin	African catfish, *Clarias gariepinus*	Juvenile	Static	96-h	0.006	Present study
	Bluegill, *Lepomis macrochirus*	-	Static	96-h	0.017	[48]
	Turbot, *Psetta maxima*	Embryo–larvae	Semi-static	96-h	0.097	[11]
	Striped bass, *Morone saxatilis*	-	Static	96-h	0.019	[48]
	Striped mullet, *Mugil cephalus*	-	Static	96-h	0.023	[48]
	Northern puffer, *Sphoeroides maculatus*	-	Static	96-h	0.034	[48]
	Threespine stickleback, *Gasterosteus aculeatus*	-	Static	96-h	0.015	[48]
	Bluegill, *Lepomis macrochirus*	Adult	Semi-static	24-h	0.0055	[49]
	Rainbow trout, *Oncorhynchus mykiss*	Adult	Semi-static	24-h	0.0019	[49]
	Common goby, *Pomatoschistus microps*	Adult	Semi-static	24-h	0.0035	[11]
	Plaice, *Pleuronectes platessa*	Adult	Semi-static	24-h	0.0017	[11]
Heptachlor	African catfish, *Clarias gariepinus*	Juvenile	Static	96-h	0.057	Present study
	Fathead minnow, *Pimephales promelas*	Fingerling	Static	96-h	0.094	[14]
	Guppy, *Poecilia reticulata*	Fingerling	Static	96-h	0.11	[14]
	Goldfish, *Carassius auratus*	Fingerling	Static	96-h	0.23	[14]
	Black bullhead, *Ictalurus melas*	Fingerling	Static	96-h	0.063	[50]
	Bluegill sunfish, *Lepomis macrochirus*	Fingerling	Static	96-h	0.013	[14]
	Rainbow trout, *Oncorhynchus mykiss*	Fingerling	Static	96-h	0.032	[50]
	Channel catfish, *Ictalurus punctatus*	Fingerling	Static	96-h	0.025	[14]
	Reader sunfish, *Lepomis microlophus*	Fingerling	Static	96-h	0.017	[50]
	Largemouth bass, *Micropterus salmoides*	Fingerling	Static	96-h	0.010	[50]

## Data Availability

Data are available for financial support of this study through National Agricultural Technology Program-2 (NATP-II), Bangladesh, project memo No: NATP-2/PIU-BARC-44/2017/1662 (54).
